# Bilateral pneumothorax in a patient with angiosarcoma of the scalp: a case report

**DOI:** 10.1186/s13256-023-03878-1

**Published:** 2023-05-01

**Authors:** Maham Vaqar, Ayesha Sharif, Nousheen Iqbal, Muhammad Irfan

**Affiliations:** Department of Medicine, Aga Khan University, Karachi, Pakistan

**Keywords:** Angiosarcoma, Pneumothorax, Case report

## Abstract

**Background:**

Angiosarcoma is a rare, vascular malignancy that arises from endothelial cells of blood vessels. This case report aims to create the awareness of its existence in the region and its mode of presentation.

**Case presentation:**

A 63-year-old Pakistani man presented to the emergency department with sudden bilateral chest pain and shortness of breath for 2 days. On examination, a scalp lesion was seen which had been increasing in size over the last 6 weeks. The lesion was 8 × 10 cm in size with an irregular border, non-tender, violet and dome-shaped in elevation on the right occipito-parietal lobe of the skull. Chest computed tomography (CT) showed multiple cystic lesions on both lungs, patchy areas of ground-glass opacities, nodules of variable sizes and bilateral pneumothorax. Bilateral tube thoracostomy was performed which provided symptomatic relief for shortness of breath. His bronchoalveolar lavage (BAL) was negative for infection. He underwent biopsy of scalp lesion which was positive for aggressive angiosarcoma.

**Conclusion:**

Bilateral spontaneous pneumothorax can be the initial manifestation of aggressive cutaneous angiosarcoma and frequently leads to respiratory failure. Early recognition is essential to prevent delay in diagnosis and management.

## Background

Angiosarcoma is a rare, vascular malignancy that arises from endothelial cells of blood vessels [1]. Angiosarcomas account for less than 1% of adult soft tissue sarcomas and most commonly originate in the head and neck regions of elderly males [2–4]. Patients with angiosarcoma have a poor prognosis with a 5-year survival rate of less than 15% [5]. Pulmonary involvement is usually due to metastasis from a primary site. This can manifest as distended cystic or solid lesions which can spontaneously rupture and lead to repeated pneumothorax. Currently, few studies have been reported on bilateral pneumothorax in metastatic scalp angiosarcoma.

In this report, we chronicle the case of a patient with primary scalp angiosarcoma presenting at an advanced stage as a scalp tumour. This article aims to create the awareness of its existence in the region and its mode of presentation for effective management. This can contribute to early detection of disease and improve prognosis.

## Case presentation

A 63-year-old Pakistani man presented to the emergency department with two days of sudden bilateral chest pain, shortness of breath and a mild cough. He had been experiencing shortness of breath for the last month, which had worsened in the last two days. His chest pain was sharp and pleuritic in nature. No hemoptysis was reported and there was no personal or family history of cancer, diabetes, or tuberculosis. He also reported a lesion on his scalp that had been gradually increasing in size for 6 weeks. A week before his presentation, he visited a dermatologist at another institution who performed a biopsy, suspecting basal cell carcinoma or angiosarcoma, but the histopathology report was still pending.

**Fig. 1:**
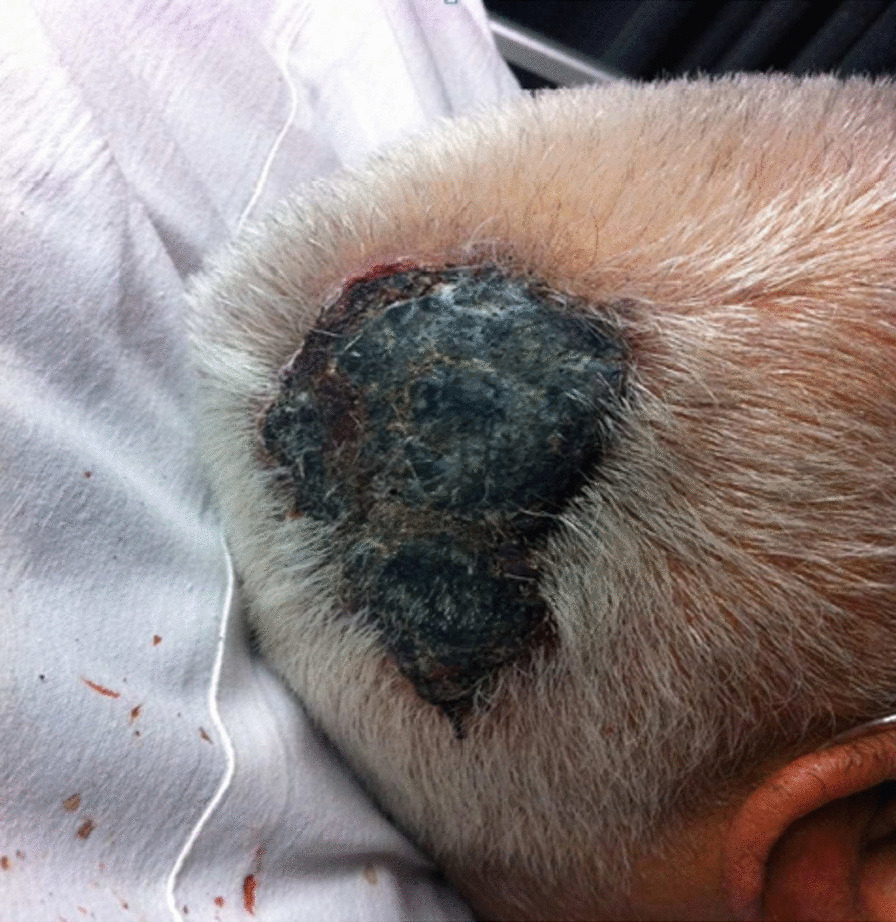
Scalp lesion (159 × 159 mm)

On admission, he appeared acutely ill. His blood pressure was 100/70 mmHg, his pulse rate was 130 beats per minute, and his body temperature was 37.6 °C. Oxygen saturation was 80% on room air and respiratory rate was 35/ min. A nonpainful, violet-colored lesion measuring 8 × 10 cm was found on the right occipito-parietal lobe of the skull, with a dome-shaped elevation and an irregular border. (Fig. 1). Breathing sounds were diminished in the bilateral lung field, and a few scattered crackles were heard in the bilateral lower zones. Other physical findings were negative.

On the day of admission, his white blood cell count was 14,500/mm^3^ with 80% neutrophils and 25% lymphocytes, the hemoglobin level was 12.5 g/dL, and the platelet count was 278,000/mm^3^. Serum biochemical values were all within the normal range. Differential diagnoses included tuberculosis which is endemic in our region. Pneumocystitis carinii pneumonia, Nocardia, fungal infection, malignancy, and Langerhans cell histiocytosis were also considered.

**Fig. 2:**
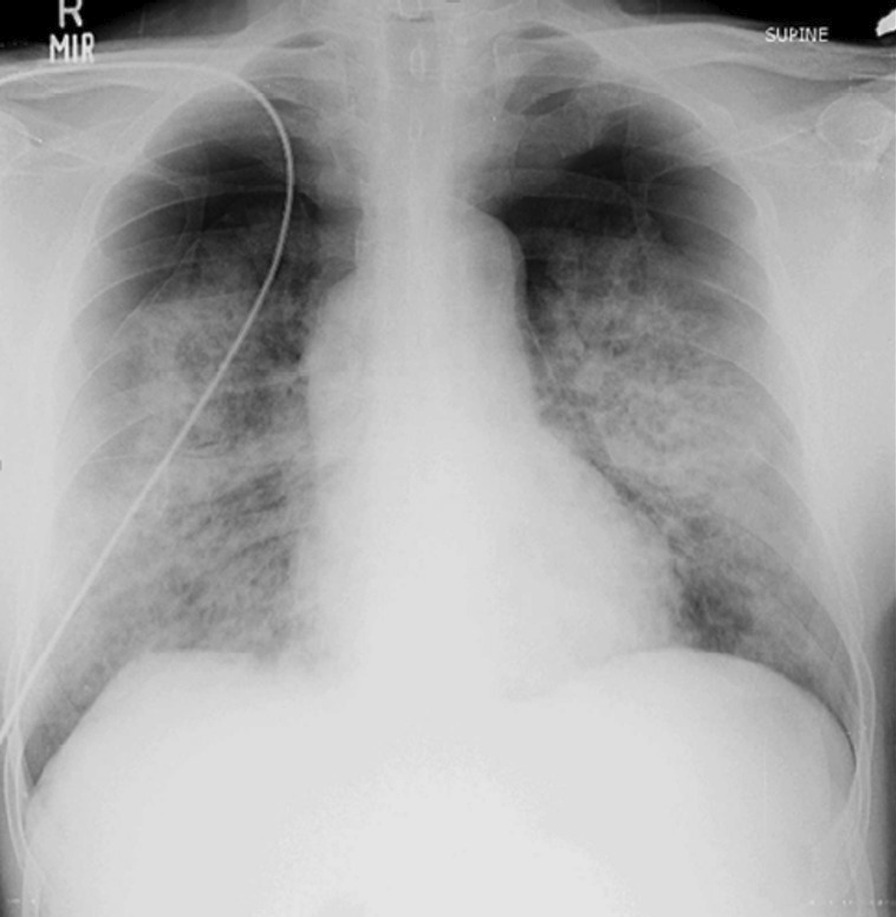
Chest X-ray showing bilateral pneumothorax

**Fig. 3:**
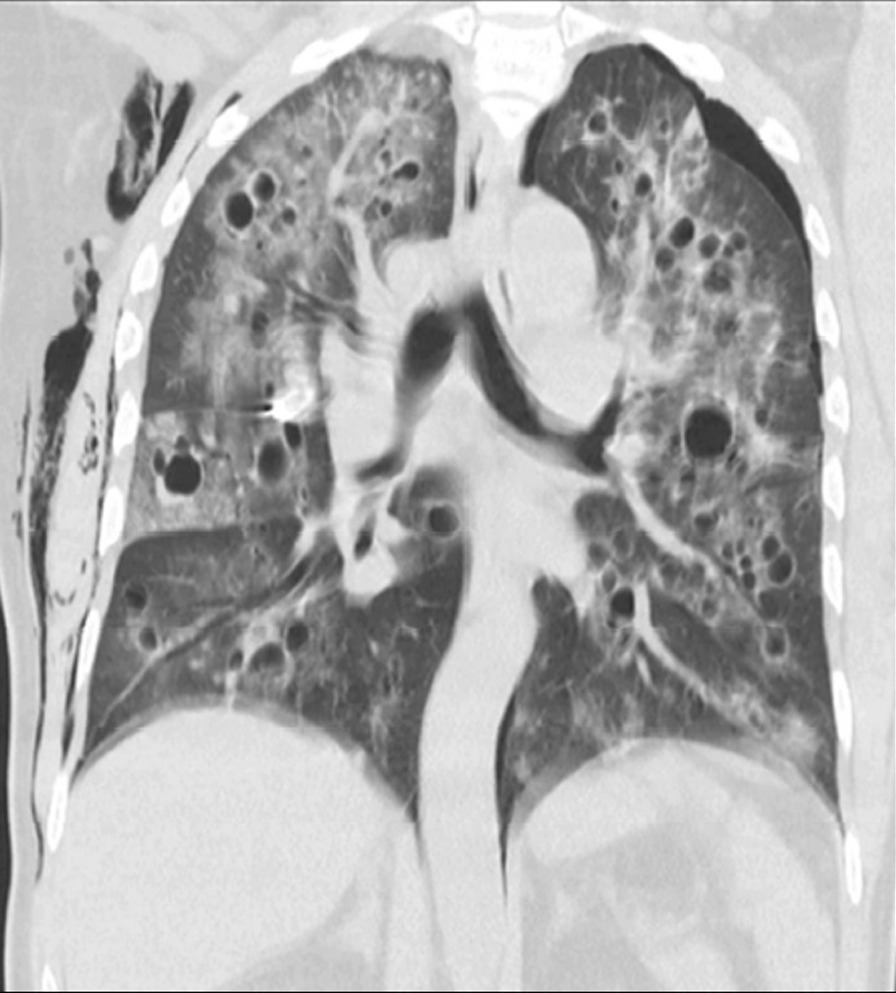
High resolution computed tomography (HRCT) Chest showing multiple cystic lesions bilaterally, patchy areas of ground glass opacities and nodules with bilateral pneumothorax

**Fig. 4:**
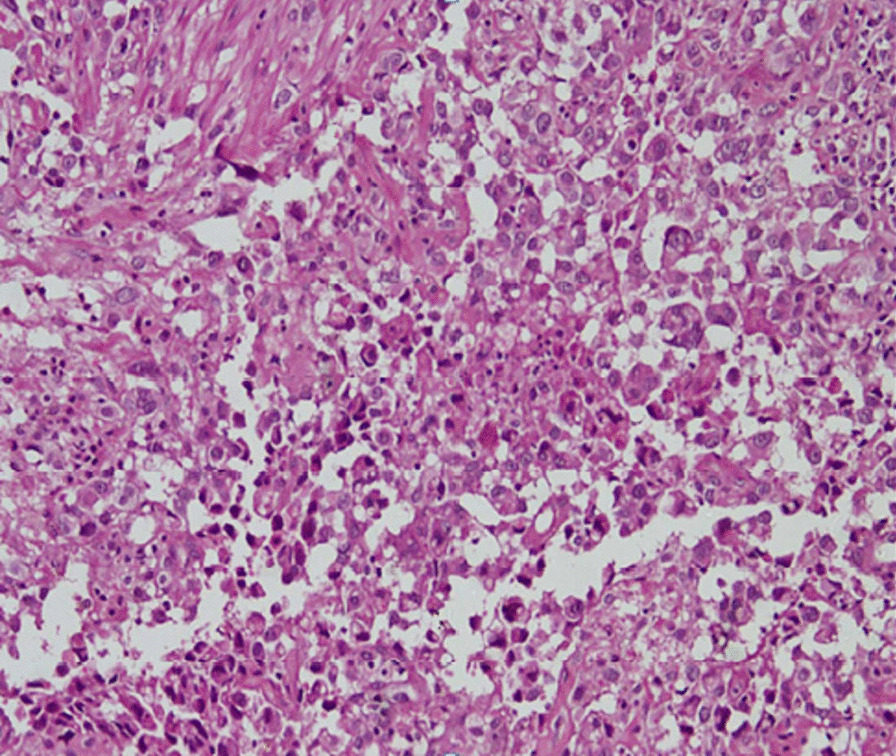
Histopathology: Section shows a neoplastic lesion arranged in sheets composed of polygonal cells with abundant eosinophilic cytoplasm, round nuclei with prominent nucleoli. The intervening areas shows irregular anastomosing channels

Chest radiography showed bilateral pneumothorax (Fig. 2) Chest computed tomography (CT) revealed multiple cystic lesions on both lungs, patchy areas of ground-glass opacities and nodules with bilateral pneumothorax (Fig. 3) Bronchoalveolar lavage (BAL) was negative for any infectious etiology, which ruled out active infections such as tuberculosis (TB), fungal infections and Nocardia. Meanwhile, histopathology results from his scalp lesion confirmed aggressive angiosarcoma (Fig. 4).

**Fig. 5:**
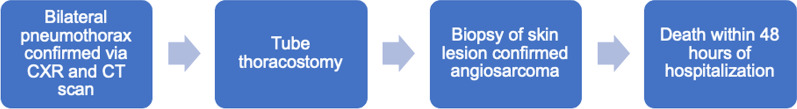
Summary of patient’s hospital course

The patient underwent bilateral tube thoracostomy and was started on broad spectrum antibiotics. Because the family refused invasive ventilation, noninvasive mechanical ventilation (NIMV) was used to assist with breathing along with broad spectrum antibiotics. Based on histopathology and CT scan findings of bilateral cystic lesion and nodules with bilateral pneumothorax on the lungs, a diagnosis of metastatic angiosarcoma was made. Given the prognosis of his disease, the patient was discharged on request because he was unwilling to undergo further treatment for the underlying metastatic disease, and he died of ARDS (acute respiratory distress syndrome) and severe respiratory failure two days later at home. An overview of the patient's hospital course is shown in Fig. 5.

## Discussion

Angiosarcoma is a rare malignant neoplasm and accounts for less than 2–3% of all cases of adult sarcomas [2]. Angiosarcoma of the scalp has an overall male–female ratio of 2:1 and has a predominance in elderly males, although it can occur at all ages [3, 4]. The clinical presentation varies among patients from dark-purple, bluish, or red lesions, which may present as a nodule or plaque [3]. In advanced cases, ulcerations, fungating masses or hemorrhagic lesions may be seen [6]. In the early stage, they can be obscured by hair on the scalp and their appearance may not raise suspicion for a malignant lesion which contributes to a delay in diagnosis. Minor trauma, such as combing hair can lead to ulceration and hemorrhage [3].

After the growth of cancer cells at the primary site, spread of cancer cells to other parts of the body through the lymphatic system often occurs, leading to metastasis. Metastasis occurs in more than half of all cases with the most common site being the lung, followed by bone and liver [7]. Patients with metastatic disease have a significantly poorer prognosis. After the appearance of lung metastases, one series reported a 4-month survival [5]. Infections such as tuberculosis or fungal infections are important differential diagnoses of lung metastasis and should be ruled out by microbiological examination of respiratory specimens such as BAL or sputum. In our case, a negative microbiological examination of bronchoalveolar lavage ruled out infections. Indeed, our 70-year-old patient with an angiosarcoma of the scalp had a very poor prognosis, particularly after the appearance of lung metastases. The scalp was reported to be the most common primary site of angiosarcoma in cases with spontaneous pneumothorax, which was also true in our case [8].

Histologic examination of the cavitary metastases to the lungs has revealed cysts which are connected to multiple tubular spaces, formed from rapidly dividing tumor cells. The weak vessel-like structure of the cavitary lung lesions is prone to breaking down and forming large and thin-walled cystic lesions [9]. These cystic changes can then present in the form of pneumothorax or hemothorax in patients with angiosarcoma [10]. An analysis of 23 autopsy cases of angiosarcoma with pulmonary metastasis found all cases of cystic metastases but one to be complicated by pneumothorax [11]. These findings are consistent with our case where the patient had bilateral cystic changes in the lung and later developed pneumothorax. The most common lesions seen on radiological imaging were thin-walled cysts (39%), nodules (39%), mixed cysts and nodules (13%), and ground-glass opacity (9%) [11].

Due to its rare presentation and low index of suspicion, the diagnosis of angiosarcoma is often delayed. Biopsy and immunohistochemical staining are the mainstay for the diagnosis of angiosarcoma with or without metastasis. Moreover, imaging using PET scan is a significant tool contributing to diagnosis, staging as well as follow-up of patients with angiosarcoma [12, 13].

Although there are no specific, well-established guidelines for the management of angiosarcoma, wide margin excision along with adjunctive radiotherapy remains the most effective treatment especially for resectable tumors [14]. Unfortunately, the majority of the tumors are at an unresectable stage at the time of diagnosis and for such patient multimodal approach using an amalgamation of radiotherapy, chemotherapy, and immunotherapy is considered. For patients with metastatic disease, chemotherapy is the principal treatment of choice. Some of the most effective chemotherapeutic agents include doxorubicin, ifosfamide and taxanes. Gemcitabine, bevacizumab, and sorafenib have also shown activity against metastatic angiosarcoma [10, 13]. Recent trials are studying therapies targeted against VEGF and tyrosine kinases, nonetheless the prognosis remains poor [15, 16].

## Conclusion

Angiosarcoma is a rare malignant soft tissue tumor with a low clinical index of suspicion. Delays in diagnosis can lead to inappropriate disease management as well as disease progression. The clinical phenotype varies, with plaques and nodules occurring with greatest frequency. Cutaneous angiosarcoma should be suspected in patients presenting with spontaneous pneumothorax with cutaneous lesions, particularly on the scalp which is the most common presenting site of angiosarcoma. Skin biopsy is imperative to confirm diagnosis**.** Unfortunately, the majority of the tumors are at an unresectable stage at the time of diagnosis and for such patients a multimodal approach using an amalgamation of radiotherapy, chemotherapy, and immunotherapy is considered. The prognosis is generally poor and therefore early recognition is essential to prevent delay in diagnosis and management.

## Data Availability

The data for this case report are located at Aga Khan University Hospital, Karachi, Pakistan.
